# Surveillance systems to monitor antimicrobial resistance in *Neisseria gonorrhoeae*: a global, systematic review, 1 January 2012 to 27 September 2020

**DOI:** 10.2807/1560-7917.ES.2022.27.18.2100917

**Published:** 2022-05-05

**Authors:** Nicholas A Medland, Ye Zhang, Praveena Gunaratnam, David A Lewis, Basil Donovan, David M Whiley, Rebecca J Guy, John M Kaldor

**Affiliations:** 1 Kirby Institute, University of New South Wales, Sydney, Australia; 2 Westmead Clinical School and Marie Bashir Institute for Infectious Diseases and Biosecurity, University of Sydney, Australia; 3 Sydney Sexual Health Centre, Sydney, Australia; 4 Faculty of Medicine, University of Queensland, Brisbane, Australia

**Keywords:** gonorrhoea, resistance, antimicrobial, surveillance, Neisseria, antibiotic, infection

## Abstract

**Background:**

Effective surveillance of antimicrobial resistance (AMR) in *Neisseria gonorrhoeae* is required for the early detection of resistant strains and to ensure that treatment guidelines are appropriate for the setting in which they are implemented. AMR in *N. gonorrhoeae* has been identified as a global health threat.

**Aim:**

We performed a systematic review to identify and describe surveillance systems targeting AMR in *N. gonorrhoeae*.

**Methods:**

We searched Medline, PubMed, Global Health, EMBASE, CINAHL, Web of Science and ProQuest databases and grey literature between 1 January 2012 and 27 September 2020. Surveillance systems were defined as the continuous, systematic collection, analysis and interpretation of *N. gonorrhoeae* resistance data. The key components of surveillance systems were extracted, categorised, described and summarised.

**Results:**

We found 40 publications reporting on *N. gonorrhoeae* AMR surveillance systems in 27 countries and 10 multi-country or global surveillance reports. The proportion of countries with surveillance systems in each of the WHO's six regions ranged from one of 22 countries in the Eastern Mediterranean and five of 54 in Africa, to three of 11 countries in South East Asia. Only four countries report systems which are both comprehensive and national. We found no evidence of a current surveillance system in at least 148 countries. Coverage, representativeness, volume, clinical specimen source, type and epidemiological information vary substantially and limit interpretability and comparability of surveillance data for public health action.

**Conclusion:**

Globally, surveillance for *N. gonorrhoeae* AMR is inadequate and leaves large populations vulnerable to a major public health threat.

## Introduction

*Neisseria gonorrhoeae* is one of the most common curable sexually transmitted infections (STI) with an estimated 78 million new gonorrhoea cases worldwide each year [1]. Since the dawn of the antibiotic era, strains of *N. gonorrhoeae* resistant to guideline-recommended antibiotics have emerged, beginning with penicillin and moving through tetracyclines, spectinomycin, fluoroquinolones and, more recently, treatment failures with macrolides and oral third-generation extended-spectrum cephalosporins [2,3]. Most guidelines now recommend injectable ceftriaxone, sometimes in combination with oral azithromycin [4,5]. However, ceftriaxone-resistant strains have been documented [6-9] and in 2018, the first cases of gonorrhoea with resistance to both ceftriaxone and azithromycin were reported [8,10].

Both gonorrhoea treatment and population exposure to antibiotics select for *N. gonorrhoeae* resistance [11]. Only surveillance can ensure that clinical guidelines match actual patterns of *N. gonorrhoeae* antimicrobial resistance (AMR) [12-14]. However, surveillance faces a number of implementation challenges because it relies on bacterial culture of specimens from people with infection. In most low- and middle-income country settings, bacterial culture and nucleic acid amplification testing (NAAT) to identify infection are not available. For this reason, clinicians treat only symptomatic patients based on identification of easily recognised symptoms and signs and without testing, an approach known as syndromic management [15]. This approach limits surveillance because asymptomatic infection cannot be identified [16]. Clinically, it also results in overconsumption of antibiotics because many of these presentations may be due to another pathogen. In high-income countries, culture-based diagnosis has been largely replaced by NAAT testing, which offers higher sensitivity and facilitates asymptomatic testing, but also reduces the availability of culture isolates for resistance testing and surveillance [17].

Tracking *N. gonorrhoeae* AMR globally is important because there is considerable geographic variation in the epidemiology of AMR, and AMR which arises in one country can become established in another through travel [18]. The World Health Organisation (WHO) Gonococcal Antimicrobial Surveillance Programme (GASP) plays a critical role in global surveillance and informing international collaboration [19,20]. Although there have been many reports of *N. gonorrhoeae* AMR methods and findings, there does not appear to have been a comprehensive report on the various approaches used for surveillance around the world. With increasing concern about the emergence of resistance, we performed a systematic review of existing surveillance systems for *N. gonorrhoeae* AMR to describe their characteristics and identify gaps to be addressed at national, regional and global levels.

## Methods

### Literature sources and search strategy

We performed a search of published literature from 1 January 2012 to 27 September 2020 using seven electronic databases (Medline, PubMed, Global Health, EMBASE, CINAHL, Web of Science and ProQuest) with search terms and variations of: gonorrhoea, *Neisseria gonorrhoea,* resistance, surveillance, sentinel, syndromic and programme. We searched records in all languages, including those other than English. The restriction on records before 2012 was to focus the review on currently active surveillance. Reference lists were hand-searched for additional records. Non peer-reviewed records were identified by searching Google and the websites of the Australian Department of Health, European Centre for Disease Prevention and Control, National Institute for Communicable Diseases (South Africa), New Zealand Ministry of Health, Public Health Agency of Canada, Public Health England (now known as United Kingdom (UK) Health Security Agency), United States Centers for Disease Control and Prevention (CDC), and the World Health Organization (WHO) using the same search string. These websites were also used to provide additional information on gonorrhoea cases by country. No restriction was made on language of publication.

### Definition of a surveillance system

We used the WHO definition of a surveillance system as ’the continuous, systematic collection, analysis and interpretation of health-related data needed for the planning, implementation, and evaluation of public health practice’ [21]. We interpreted the ‘continuous, systematic’ aspect broadly, to include ongoing data collection repeated at regular intervals but not including one-off publication of data.

### Eligibility criteria

Inclusion criteria were: (i) the publication had sufficient detail about methods to determine that the system described met the definition of surveillance system, (ii) detection of AMR in *N. gonorrhoeae* was reported as the outcome of interest and (iii) the findings were published on or after 1 January 2012. Exclusion criteria were: (i) one-off studies without an ongoing data collection component described in the methods or presented in the results and (ii) studies concerned with specific clinical or microbiological features of *N. gonorrhoeae* AMR, rather than surveillance. Where more than one article comprehensively described a particular surveillance system, only the most recent was included. Where two or more articles described non-overlapping components of a surveillance system, both were included. Although surveillance systems, as defined above, were the focus of the review, we also included reports from WHO GASP (referred to as GASP reports) for reference and comparison.

### Study selection

Articles retrieved from database searches were imported into EndNote and duplicates removed. Screening was performed by applying exclusion criteria to the title and full abstracts. Full-text articles were then downloaded for eligibility assessment. Screening was performed in parallel and independently by NM and PG, or NM or YZ, with disagreements discussed and resolved by consensus.

### Data extraction and analysis

We developed, piloted and applied a standardised extraction tool based on WHO recommendations for necessary components of national antimicrobial resistance surveillance systems [22]. These components included the type of surveillance and the elements of the surveillance system such as: antimicrobial agents chosen for susceptibility testing, testing method, number of samples, timeframe, population, location and type of collection site (See Supplementary Table S1: Antimicrobial resistance surveillance system core components checklist). Extracted data were stored in an Excel (Microsoft Corp.) database.

Surveillance systems were categorised as comprehensive if they include all types of clinical sites and laboratories or sentinel if they include specific selected clinical or laboratory sites based on specified criteria. Surveillance systems were categorised as national if they covered the entire country or greater than 50% of states or provinces, or subnational with less than 50% of states or provinces.

Some records described multi-country programmes but did not contain enough detail on individual country systems to meet eligibility criteria, most notably the GASP reports. These were included for reference but not in the descriptive analysis.

The findings of this review are reported in accordance with the PRISMA Statement [23].

## Results

**Figure 1:**
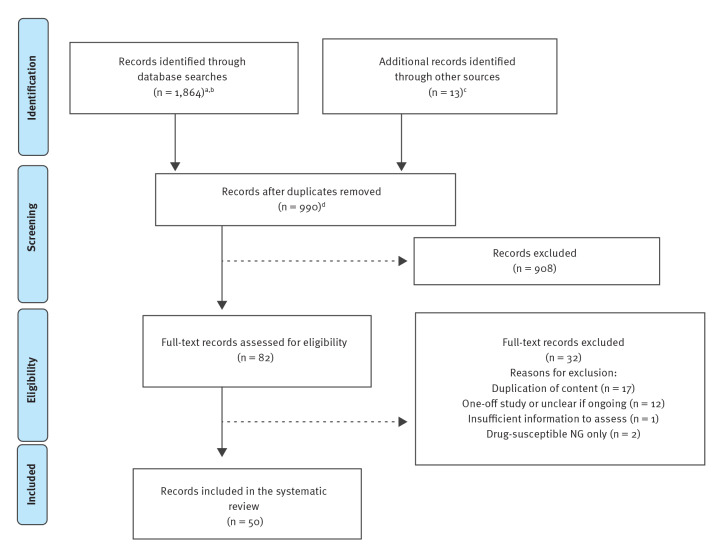
PRISMA flowchart for search strategy and literature review of surveillance systems, worldwide, to monitor antimicrobial resistance in *Neisseria gonorrhoeae*, 1 January 2012–27 September 2020

A total of 1,864 records were identified through database searches and 13 through a grey literature search. Screening after removal of 990 duplicates identified 82 records for full-text assessment, of which 50 were eligible for inclusion (Figure 1). Forty records described 32 country-level *N. gonorrhoeae* AMR surveillance systems in 27 countries, while 10 records described four regional WHO GASP reports and two global WHO GASP reports (Table 1).

NG*: Neisseria gonorrhoea*.

^a^ Medline, PubMed, Global Health, EMBASE, CINAHL, Web of Science, ProQuest

^b^ Search keywords were ‘gonorrhea’ OR ‘gonorrhoea’ OR ‘*Neisseria gonorrhoeae’* AND ‘drug resistance’ OR ‘drug resistance, microbial’ OR ‘drug resistance, bacterial’ OR ‘multidrug resistance’ OR ‘antibiotic resistance’ AND ‘population surveillance’ OR ‘surveillance’ OR ‘sentinel surveillance’ OR ‘syndromic surveillance’ OR ‘drug surveillance programme’.

^c^ Reference lists and grey literature.

^d^ No restriction was made on language of publication. All records identified were screened using an English language title and abstract, which was available in all cases. All records assessed for eligibility were in English.

**Table 1:** Characteristics of *Neisseria gonorrhoeae* antimicrobial resistance surveillance systems, worldwide, 1 January 2012−27 September 2020 (n = 32)

Country	Included report(s)	Surveillance system name^a^	Est.	System type^b^	Coverage^c^	Data sources^d^	Number of isolates	Timeframe
**WHO African Region**								
Ghana	[24]	(United States) Armed Forces Health Surveillance Centre (AFHSC) Network	NA	Other	Subnational	Military clinics, hospitals	13	2012–13
Kenya	[25]	NA	NA	Other	Subnational	Sex worker outreach	238	2012–15
South Africa	[26,27]	NA	2005	Sentinel	Subnational	Primary care	4,224^e^	2008–15
Côte d'Ivoire	[29]	NA	NA	Sentinel	National	Sexual health	212	2014–17
Zimbabwe	[28]	NA	NA	Sentinel	Subnational	Primary care	102	2015–16
**WHO Region of the Americas**								
Argentina	[30]	Gonococcal antimicrobial surveillance system	1983	Other	National	Hospitals	1,987	2009–13
Brazil	[31]	NA	NA	Sentinel	National	Sexual health, hospitals	550	2015–16
Canada	[32,33,39]	National Surveillance of Antimicrobial Susceptibilities of *N. gonorrhoeae*	1985	Comprehensive	National	NA	4,538	2016
United States	[34-37]	Gonococcal Isolate Surveillance Project (GISP)	1986	Sentinel	National	Sexual health	5,160	2018
	[37,38]	Enhanced Gonococcal Isolate Surveillance Project (eGISP)	2017	Sentinel	Subnational	Sexual health	16,842^f^	2017–18
	[37]	Strengthening the US. Response to Resistant Gonorrhoea (SURRG)	2016	Sentinel	Subnational	Sexual health		
**WHO Eastern Mediterranean Region **								
Morocco	[40]	NA	1998	Sentinel	National	Primary care	72	2009
**WHO European Region **								
Austria	[41]	NA	NA	Other	National	Sexual health	3,584	2010–14
Belarus	[42]	NA	2009	Sentinel	Subnational	Sexual health	193	2010–13
United Kingdom	[43,44]	Gonococcal Resistance to Antimicrobials Surveillance Programme (England and Wales)	2000	Sentinel	Subnational	Sexual health	1,284	2016
	[45,46]	Second Generation Surveillance System (England and Wales)	NA	Comprehensive	National	NA	17,099	2016
Scotland	[47,54]	Gonococcal Antibiotic Surveillance in Scotland (GASS)	NA	Comprehensive	National	Primary care, Sexual health	3,168	2018
France	[48]	Rénago - National Gonorrhoea Network	NA	Sentinel	National	NA	8,649	2001–12
Germany	[49]	Gonococcal Resistance Network (GORENET)	2014	Sentinel	National	NA	1,654	2014–15
Italy	[50]	NA	NA	NA	National	Sexual health	1,688	2009–16
Netherlands	[51]	NA	2007	Other	Subnational	Sexual health	11,678	2007–15
Russia	[52]	Russian Gonococcal Antimicrobial Surveillance Programme (RU-GASP)	2004	Other	National	Sexual health	5,038	2005–16
Switzerland	[53]	NA	NA	Other	Subnational	NA	318	1990, 2000–12
**WHO South-East Asia Region **								
India	[55]	NA	NA	Other	Subnational	Sexual health	124	2013–16
Nepal	[56]	NA	1998	Other	National	Not specified	181	1999–2012
Thailand	[57]	Enhanced Gonococcal Antimicrobial Surveillance Programme (E-GASP)	2015	Sentinel	Subnational	Sexual health	590	2015–16
**WHO Western Pacific Region**								
Australia	[64]	Australian Gonococcal Surveillance Programme	1979	Comprehensive	National	All sites	9,668	2019
China	[58]	China Gonococcal Resistance Surveillance Programme (Mainland China)	1987	Sentinel	Subnational	Sexual health	3,849^g^	2013–16
	[59]	Hong Kong SAR	NA	Other	National	Sexual health	947	2010
Japan	[60]	NA	NA	Other	National	Medical institutions	2,471	2000–15
Korea	[61]	NA	NA	Other	National	All sites	210	2011–13
New Zealand	[62,63]	NA	NA	Comprehensive	National	NA	667	2014–15
**Multi-country Gonococcal Antimicrobial Surveillance Programmes**								
Europe	[65-68,70]	Euro-GASP	2009	Sentinel	Supp.^h^	Supp.^h^	NA	2010–18
Latin America and the Caribbean	[71]	LAC-GASP	1990	Other	Supp.^h^	Supp.^h^	NA	1990–2011
South-East Asia	[72]	SEAR-GASP	1997	Other	Supp.^h^	Supp.^h^	NA	2009–12, 2016
Western Pacific	[73]	WPR-GASP	1992	Other	Supp.^h^	Supp.^h^	NA	2016
Global GASP	[88,89]	NA	1990	Mixed	Supp.^h^	Supp.^h^	NA	2009–14, 2016

Est.: year established; GASP: gonococcal antimicrobial surveillance programmes; NA: not available in cited publication(s); SAR: special administrative region; Supp.: supplementary materials.

^a^ The names of surveillance systems were listed if available.

^b^ System type: comprehensive: includes all healthcare providers and laboratories; sentinel: selected sites chosen on specified criteria; other: selected sites by criteria not specified.

^c^ Coverage: national: country-wide of greater than 50% of all states or provinces; subnational: less than 50% of states and provinces.

^d^ Data sources: primary care including general practice, community health centres and clinics, sexual health services including clinics and networks for diagnosis and treatment of sexually transmissible infections, genitourinary medicine departments, dermato-venereology services.

^e^ From two geographic areas only.

^f^ Samples across Gonococcal Isolate Surveillance Project (GISP), Enhanced Gonococcal Isolate Surveillance Project (eGISP) and Strengthening the US. Response to Resistant Gonorrhoea (SURRG) [37].

^g^ From seven (of nine) provinces only.

^h^ See Supplementary materials for description of individual country components of multi-country surveillance reports.

Of 27 individual countries with included surveillance systems, five were in the WHO African Region (n = 54 countries) [24-29], four in WHO Region of the Americas (n = 41 countries) [30-39]; one in the WHO Eastern Mediterranean Region (n = 22 countries) [40], nine in the WHO European Region (n = 53 countries) [41-54], three in the WHO South-East Asia Region (n = 11 countries) [55-57], and five in the WHO Western Pacific Region (n = 27 countries) [58-64]. See Table 1 and Supplementary Table S2a and Table S2b, which provide an overview of surveillance systems.

WHO GASP and the European Centre for Disease Prevention and Control have published reports which include data from 27 countries in the European Union/European Economic Area [65-70]. Although these may be considered ongoing surveillance systems, the description of each individual country level system did not meet inclusion criteria. In addition, WHO GASP reports included 23 Latin American and Caribbean countries [71], six South-East Asian Countries [72] and 12 Western Pacific countries [73]. See Table 1 and Supplementary Table S2 for additional surveillance details.

### Comprehensiveness and national coverage

**Figure 2:**
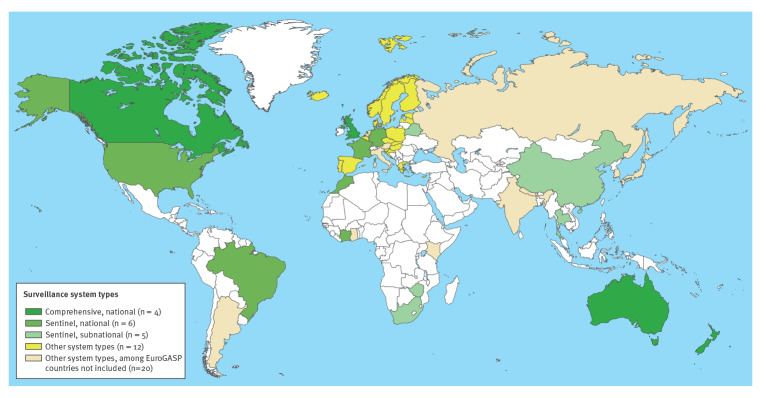
Types of country-level *Neisseria gonorrhoeae* antimicrobial resistance surveillance systems, worldwide, 1 January 2012−27 September 2020 (n = 47 countries)

Globally, four countries had gonorrhoea AMR surveillance systems which were both comprehensive and national in that they aimed to cover all culture-evaluable gonorrhoea diagnoses and covered more than 50% of jurisdictions: Australia, Canada, New Zealand and the UK. Of the remaining 23 countries, coverage was national in 13 and subnational in 10, in that they reported data from less than 50% of states or provinces. Among those 23 countries, the selection criteria for sentinel sites were reported in 12 and not reported in the remaining 11. See Table 1 and Figure 2.

GASP: gonococcal antimicrobial surveillance programmes.

Of 27 countries in the Euro-GASP report [66], seven are included in this review (see Table 1) and marked here by system type. Although the remaining Euro-GASP 20 countries (beige) could be considered to have ongoing surveillance, the description of each individual country level system did not meet inclusion criteria for separate inclusion in this review.

Altogether, four countries have *N. gonorrhoeae* AMR surveillance systems which are national and comprehensive, six countries have national, sentinel surveillance systems, five countries have subnational sentinel surveillance systems, seven countries have systems which are national but neither comprehensive nor sentinel, five countries have other systems which are neither national nor comprehensive nor sentinel; at least 148 countries did not have evidence of systematic, ongoing *N. gonorrhoeae* AMR surveillance. See Table 1 and Figure 2.

### Population, sampling methods and clinical site

Ten of the country-level systems reported data from symptomatic patients alone, including five from males only and one from female sex workers only. Nine country-level systems used protocols requiring antimicrobial susceptibility testing of all culture-positive cases presenting within a specified time frame; three used convenience sampling, and 19 did not specify the sampling method. Nine country-level systems specified that sample collection took place in STI clinics alone, while four also included other health services such as hospitals, 10 used other health services alone, and nine did not specify (Table 2 and Supplementary Table S2: country surveillance systems). Data on population, sampling and clinical site collection from GASP reports, where available, are also included in Supplementary Table S2.

**Table 2:** Attributes of country-level *Neisseria gonorrhoeae* antimicrobial resistance surveillance systems (aggregated), worldwide, 1 January 2012−27 September 2020 (n = 32 systems in 27 countries)

Attributes	Country-level systems (n = 32)			
System type^a^	Comprehensive (n = 5)	Sentinel (n = 14)	Other (n = 12)	NS (n = 1)
Geographical coverage^b^	National (n = 19)	Subnational (n = 12)	NS (n = 1)	
Population^c^	Symptomatic (n = 10)	Laboratory (n = 14)	NS (n = 8)	
Sampling	Consecutive (n = 9)	Convenience (n = 3)	Mixed (n = 1)	NS (n = 19)
Where isolates are collected	STI clinics (n = 9)	Other services (n = 10)	Both (n = 4)	NS (n = 9)
Anatomical sites^d^	Yes (n)	No (n)	NS (n)	
Male anogenital	25	1	6	
Male non-anogenital	13	13	6	
Female anogenital	21	5	6	
Female non-anogenital	13	12	7	
Patient characteristics^d,e^	Yes (n)	No (n)	NS (n)	
Age	21	3	8	
Sex	21	4	7	
Site of infection	11	11	10	
Other demographic information^f^	8	15	9	
Behavioural	13	12	7	
Clinical	12	13	7	

NS: Not specified.

^a^ System type: comprehensive: includes all healthcare providers and laboratories; sentinel: selected sites chosen on specified criteria; other: selected sites by criteria not specified.

^b^ Coverage: national: country-wide of greater than 50% of all jurisdictions; subnational: less than 50% of states and provinces.

^c^ Population: symptomatic: surveillance specimens collected from patients presenting with gonorrhoea infection syndromes; laboratory: specimens collected from laboratory confirmed diagnoses.

^d^ For attributes of anatomical sites and patient characteristics, the n number specified under categories Yes or No refers to the number of surveillance systems of the total 32 that do or do not collect data on respective variables. NS indicates that the system does not specify collection of data.

^e^ Patient characteristics: patient characteristics information could include sociodemographic characteristics (age, sex and other), site of infection, behavioural information (e.g. sexual history), and clinical information (e.g. co-infections).

^f^ Other demographic information: other demographic information could include area of residence and sexual orientation.

### Anatomical site of collection and patient characteristics

Male anogenital specimens were collected by 25 country-level systems, female anogenital specimens by 21 and 14 collected both. Collection of non-anogenital (pharyngeal) specimens was reported by 13 (Table 2 and Supplementary Table S3: anatomical site, reported cases and data collection variables, along with data from GASP reports, where available).

Most country-level systems reported data by age (n = 21) and sex (n = 21), while a third reported by site of infection (n = 11), other demographic information such as area of residence (n = 8), behavioural information such as sexual history (n = 13) and 12 reported clinical information such as co-infections. For six country-level systems, information was insufficient to ascertain what characteristics were collected (Table 2 and Supplementary Table S3). Data on patient characteristics from GASP reports, where available, are also included in Supplementary Table S3.

### Numbers of isolates tested

The time period over which reported numbers of isolates were tested varied from less than 1 up to 12 years and the total number of isolates ranged from 13 in Ghana (over 2 years) [24] to 17,099 in England and Wales (over 1 year) [45,46] with a median of 1,654. Based on available data from 16 country-level systems, the proportion of all gonorrhoea diagnoses which resulted in an isolate included in surveillance ranged from 1% to 62% (median: 22), although there were differences in how these numbers were reported (Table 1 and Supplementary Table S3 anatomical site, reported cases and data collection, including data on numbers of isolates tested from GASP reports, where available).

### Location of laboratories

Susceptibility testing was performed in single, central laboratories for 16 country-level systems and decentralised in 14 with two unspecified. Some European Region GASP participating countries (Euro-GASP) used laboratories in Sweden or the UK [65-68,70]. Within Euro-GASP, eight were centralised and 19 decentralised [70], while for the South East Asia Region GASP four were centralised and two decentralised [72] (Supplementary Table S4: Laboratory procedures used in included surveillance systems).

### Antimicrobial susceptibility testing

Sixteen country-level systems used agar dilution, 18 used E-test, and three used both laboratory methods to determine minimum inhibitory concentrations. Five countries reported using disk diffusion. Fifteen country-level systems reported use of a beta lactamase test, while 10 mentioned the use of NG-MAST typing and two reported molecular testing for antimicrobial resistance testing (Supplementary Table S4: Laboratory procedures used in included surveillance systems including data on antimicrobial susceptibility testing methods from GASP reports, where available).

Seventeen country-level systems used Clinical and Laboratory Systems Institute (CLSI) criteria to interpret breakpoints and 10 used EUCAST guidelines, with six using other guidelines or not specifying the criteria used [74,75]. Virtually all country-level systems reported testing for susceptibility to ceftriaxone (n = 27 countries), ciprofloxacin (n = 26), azithromycin (n = 25), and penicillin (n = 22) with smaller proportions for tetracycline (n = 20), spectinomycin (n = 16), cefixime (n = 19) and other drugs such as cefpodoxime (n = 10) (Supplementary Table S4: laboratory procedures).

## Discussion

This systematic review of *N. gonorrhoeae* AMR surveillance systems takes a perspective that is both global and methodological. We have found that in most WHO regions, information on adequate systems was only available from a few countries. We also found major gaps in coverage, comprehensiveness and representativeness as well as wide variation in the methodology. These gaps indicate serious vulnerability in countries' capacity to detect, accurately monitor and respond to *N. gonorrhoeae* AMR and represents a global health risk in a world connected through travel.

Most striking is the lack of surveillance in low- and middle-income countries with high burden of disease. For example, the WHO African and the Western Pacific Region have the highest gonorrhoea prevalence and incidence [1,76], but we were only able to retrieve information on systems in place for five of 54 WHO African Region countries (Côte d'Ivoire, Ghana, Kenya, South Africa and Zimbabwe) and five of the higher income countries in the WHO Western Pacific Region (Australia, China, Japan, Korea, New Zealand). For all remaining countries, including several with populations greater than 100 million inhabitants, such as Indonesia, Nigeria, Philippines and Vietnam, there were no publications of systematically collected respective data. Also, some countries with very large populations have surveillance systems which report on very small numbers of specimens, e.g. India with a population greater than 1 billion reported only 124 specimens included in surveillance over 4 years [55].

The paucity of surveillance represents a global threat and not just to countries without surveillance. For example, cases of extensively drug-resistant (XDR) gonorrhoea have only been detected in high income countries with surveillance systems and are predominantly associated with travel to countries with limited surveillance [6,10]. Globally, much or indeed most travel for tourism, employment, study and business is likely to occur between countries identified in this review with limited surveillance [77]. As a result, both local transmission and international dissemination of resistance may be entirely undetected.

Where surveillance is not national and comprehensive, many factors may reduce representativeness or the extent to which data collected are reflective of target populations. This has important consequences for whether resistance is detected by surveillance. Ten countries have systems which only sample symptomatic patients who are more likely to be heterosexual men, and less likely to be women who bear the greatest burden of disease and men who have sex with men who have the highest incidence [78]. Resistance patterns may differ by sex and behaviour where gonococcal clones circulating in networks of men who have sex with men are distinct from those found in heterosexual men and women [79]. Many countries only collect specimens from specialist services which may see more patients who been exposed to antibiotics than in primary care [80].

To fill the gaps, WHO has developed standardised gonococcal AMR surveillance protocols, implemented with critical support of GASP regional collaborating centres [76,81], but coverage remains low in many regions. The WHO GASP plays a critically important role in standardising, compiling and presenting data from countries and regions where data would otherwise be unavailable or inaccessible [19]. We searched publications from 2012 to focus our review on currently active surveillance and direct attention to future surveillance needs. It is important to note that many countries with good laboratory capacity were engaged in gonococcal AMR testing before this time. In particular, WHO GASP was established in 1990 and has been collecting and disseminating resistance data and providing standards for laboratory capacity from that time.

For many countries, comprehensive, national systems involving high quality epidemiological data collection and quality assured laboratory testing are unlikely to be affordable with current levels of resourcing, but remain the gold standard. Countries can nevertheless aim to identify epidemiologically relevant populations at higher risk of STI and therefore more likely to be exposed to antimicrobial resistant *N. gonorrhoeae*, e.g. sex workers, travellers, men who have sex with men, and ensure that they are appropriately represented and recognised in sampling. Surveillance should include both asymptomatic and symptomatic infection, by identifying people with infection through NAAT testing as well as clinical presentations. Sampling must also incorporate adequate coverage of regions to ensure that geographical diversity can be detected, particularly in regions with increased STI vulnerability. This can be achieved either with decentralised laboratory capacity (with local specimen processing) or specialised transport to a central laboratory.

Barriers to adequate surveillance include lack of funding, prioritisation within national health agendas, human resources, education and training, as well as limited clinical and laboratory infrastructure and the technical complexity of culture-based systems [82]. The current global approach to *N. gonorrhoeae* AMR surveillance is based on testing specimens for susceptibility using bacterial culture, which is the gold standard but faces major limitations [83]. Firstly, identifying cases for sampling is difficult because most infections in women and men who have sex with men are asymptomatic and much of the world lacks access to the nucleic acid amplifications tests (NAAT) required for screening asymptomatic patients. Secondly, the fastidious nature of *N. gonorrhoeae*, and its vulnerability to degradation during transport makes culture technically challenging and, in much of the world, restricts surveillance to sites proximal to national reference laboratories [84].

Molecular resistance testing to supplement conventional culture-based methods is a promising approach as specimens can be stored inexpensively and transported and processed centrally [85]. For example, molecular assays in remote parts of the Northern Territory of Australia have produced comparable estimates of resistance compared to culture-based methods [86]. Research into cost-effectiveness of molecular testing in both low- and high-income countries and in different types of clinical settings is required. Because molecular testing can only detect known genetic markers of resistance, it needs to be supported by research to rapidly produce targets for newly identified or emergent resistant strains. Global expansion of molecular testing for *Mycobacterium tuberculosis* and more recently severe acute respiratory syndrome coronavirus 2 (SARS-CoV-2) presents opportunities to address this [87].

Aetiological testing of asymptomatic patients via NAAT testing could be made available in many settings where it is currently not being used by making use of diagnostic systems used for other diseases, such as the GeneXpert platform widely accessible for tuberculosis. Incorporation of NAAT testing has the potential to increase the scope of populations and locations where surveillance can occur. However, even with NAAT testing, the requirement for local gonococcal culture capacity is a substantial obstacle in most regions within the majority of countries, and will require ongoing technical and resource support to implement. Research to determine if other forms of AMR testing, in particular molecular testing, can augment and extend the reach of surveillance is urgently required.

This review has limitations which should be considered in interpreting its findings. Firstly, many countries contributing data which are published in WHO GASP reports may have country level surveillance systems which were not separately published or described and hence not accessible to this review [21]. Secondly, some countries may have published data from surveillance systems but the methods were not sufficiently detailed to meet all inclusion criteria or to determine that they were not one-off studies. As a result, we may have underestimated coverage of surveillance systems in some regions. Thirdly, data from country-level surveillance systems may have been published before 2012 and then not between 2012 and 2020. As a result, systems which are active but have not recently published may not have been included and we may have underestimated coverage of surveillance systems in some regions. However, it should be noted that regular dissemination of data, though not necessarily by publication, is a criterion of a surveillance system.

### Conclusions

Our review underscores that, globally, surveillance of gonorrhoea AMR is inadequate. Too few countries have surveillance systems and too few systems are adequate. Without adequate surveillance, countries lack basic information on which to base guidelines to limit treatment failure in the population. Without adequate surveillance, countries’ capacity to detect or respond to resistance when it occurs is absent or limited. However, the capacity-building requirement in many low- and middle-income countries is not to be understated. Simplified and streamlined systems are much more likely to be successfully implemented.

Moving forward, surveillance systems worldwide should be strengthened to effectively monitor and address AMR in *N. gonorrhoeae*. All countries, will bear the burden of the failure to prevent, detect and respond to *N. gonorrhoeae* AMR, irrespective of where it arises. Efforts to strengthen surveillance systems should be integrated with antibiotic stewardship initiatives and increasing access to diagnostic technology where it is not currently available.

## Supplementary Data

484000 Supplement
