# Supplementary material for: Surveillance systems to monitor antimicrobial resistance in Neisseria gonorrhoeae: a global, systematic review, 1 January 2012 to 27 September 2020
Source: Euro Surveill. 2022 May 5;27(18):2100917. doi: 10.2807/1560-7917.ES.2022.27.18.2100917 (PMC9074396; doi:10.2807/1560-7917.ES.2022.27.18.2100917)
Supplement: Supplement [file 21-00917_MEDLAND_Supplement.pdf]

This supplementary material is hosted by *Eurosurveillance* as supporting information alongside the article "Surveillance systems to monitor antimicrobial resistance in *Neisseria gonorrhoeae*: a global, systematic review" on behalf of the authors, who remain responsible for the accuracy and appropriateness of the content. The same standards for ethics, copyright, attributions and permissions as for the article apply. Supplements are not edited by *Eurosurveillance* and the journal is not responsible for the maintenance of any links or email addresses provided therein

Supplementary Table S1 Antimicrobial Resistance Surveillance System Core Components Checklist: Data extracted for review (WHO 2005) (1)

**Field**	**Data extracted**
Bibliographic information	Title, author, year of publication
Country or region covered	For example, Thailand (country) or South East Asia (region)
Type of surveillance	Comprehensive (all health-care providers/labs), sentinel/targeted (sites chosen based on specified criteria) or other (not clear how sites were chosen)
Surveillance system components	
Population under surveillance	Male or female; symptomatic or laboratory-confirmed gonorrhoea patients
Geographic coverage	National (country-wide) or sub-national (selected states or provinces within a country). Systems were classified as national if 50% or more of all states or provinces in country were included.
Sampling of patients	Convenience, consecutive, other
Period of time over which data are collected	For example, 2011 to 2015
Where isolates are collected	STI clinics, other health services
Number and percentage of total gonorrhoea cases with isolates tested for antimicrobial resistance	Number and percentage of total reported gonorrhoea cases with isolates tested for antimicrobial resistance
Patient data collected	Demographic, epidemiological, clinical
Anatomical site of specimens	Male anogenital (urethral, rectal), female anogenital (cervical, rectal, urethral, vaginal), male or female non-anogenital (e.g. pharyngeal)
Type of laboratory testing performed	AGAR dilution, E-test, disk diffusion, b-lactamase production, NG-MAST typing, molecular typing
Type of laboratory system	Centralised (only one central laboratory contributes data to the surveillance system) or decentralised (data from participating laboratories sent to a central site for collation and analysis)
Drugs tested for antimicrobial-resistant gonorrhoea	Cefixime, ceftriaxone, azithromycin, ciprofloxacin, penicillin, tetracycline, spectinomycin, other
Guidelines used as reference in determining minimum inhibitory concentration (MIC) breakpoints or susceptibility	Clinical and Laboratory Standards Institute (CLSI), European Committee on Antimicrobial Susceptibility Testing (EUCAST), Calibration, Dichotomy and Susceptibility (CDS) test or other

Supplementary Table S2a System type, coverage, target population, sampling frame and collection sites for included country surveillance systems.

Country/Region	System Type	Coverage	Population	Sampling	Where isolates are collected
**WHO Africa Region**					
Ghana (2)	Other	Subnational – 3 outpatient military clinics in Takordia (port city), 37 military hospitals in Accra (capital city)	Patients aged 12 years or older who meet symptomatic NG case definition (i.e discharge)	Convenience	Military clinics and hospitals
Kenya (3)	Other	Subnational – 1 site in Nairobi (capital city)	Women with vaginal/cervical discharge	NA	Sex Worker Outreach Project clinic
South Africa (4, 5)	Sentinel	Subnational – more than one sentinel clinic, in at least two sites – Johannesburg and Guateng province, precise locations unknown	Patients with urethral or vagina/cervical discharge	Consecutive	Primary health care services
Zimbabwe (6)	Sentinel	Subnational – 2 health centres in Harare and 2 in Bulawayo (major cities), 1 health centre in Beit Bridge (close to border with South Africa)	Men 18 years or older with visible urethral discharge, who provided consent	NA	Health centres
Côte d'Ivoire (7)	Sentinel	National- 8 sentinel sites in the Abidjan, and 6 sentinel sites across country representing a national STD network	Patients with gonorrhoea, including asymptomatic, with a focus on symptomatic men and their sexual partners		Not specified
**WHO Americas Region**					
Argentina (8)	Other	National – 70 hospitals in 20 out of 24 provinces in Argentina	NA	NA	Hospitals
Brazil (9)	Sentinel	National – 7 sites that represent all 5 regions (2 South East, 2 South, 1 North, 1 North-East, 1 Center-West)	Men 18 years of older with urethral discharge, excluded if treated with antibiotics in the past week or partner of index case	NA	Dermatology and STD clinics, hospitals
Canada (10, 11)	Comprehensive	National	NA	NA, (systematic in Alberta)	Not specified
USA (12-15)	Sentinel	Subnational - 27 clinic sites across 24 state and city health departments weighted towards western states/port cities	First 25 men presenting each month with gonococcal urethritis	Consecutive	STD clinics
USA (eGISP)(15, 16)	Sentinel	Subnational -12 clinic sites across 9 state and city health departments	First 25 men and 25 women each month: urethral/urine, pharyngeal, rectal, vaginal and/or endocervical sites.	Consecutive	STD clinics
USA (SURRG)(15)	Sentinel	Subnational – 8 clinic sites across 8 state and city health departments	Extend to recruit community non-STD clinics doing STD testing of patients to collect specimens for GC culture testing	Consecutive	STD clinics and non-STD clinics (e.g family planning clinics and emergency department)
WHO Eastern Mediterranean Region					
Morocco (17)	Sentinel	National - five cities situated in north, central, west and south Morocco	Men with urethral discharge	NA	Basic health services
**WHO Europe Region**					
Austria (18)	Other	National – 40 sites across 8 states	NA	NA	Diagnostic centers established for surveillance
Belarus (19)	Sentinel	Subnational – 1 in Minsk (capital), 1 in Mogilev Regional, 1 in Vitebsk Regional (regions)	Patients with culture positive gonorrhoea	Mainly consecutive	Dermato-venereological dispensaries
England and Wales (GRASP) (20, 21)	Sentinel	Subnational - 23 English and 2 Welsh Genitourinary Medicine (GUM) clinics	Any patient with gonorrhoea presenting between July and September each year	Consecutive for 3/12	GUM clinics
England and Wales (SGSS) (22, 23)	Comprehensive	National	NA	NA	Not specified
France (Rénago only) (24)	Sentinel	National – 133 laboratories across metropolitan France	Patients referred by doctor who have received a microbiological NG diagnosis	NA	Not specified
Germany (25)	Sentinel	National – 23 laboratories chosen based on catchment area and number of NG tests per quarter	NA	NA	Not specified
Italy (26)	Not available	National – 14 collaborating laboratories in 12 cities (6 in the North, 3 in Centre, 3 in the South)	NA	NA	STD clinics

Country/Region	System Type	Coverage	Population	Sampling	Where isolates are collected
Netherlands (27)	Other	Subnational – 19 of a total 25 STI centres in the Netherlands	All patients where antimicrobial susceptibility data were available	NA	STI clinic
Russia (28)	Other	National – between 12 to 46 dermato-venereological dispensaries across Russia surveyed each year	Patients with culture positive gonorrhoea	Mainly consecutive	Dermato-venereological dispensaries
Scotland (22, 23)	Comprehensive	National – all referred isolates tested at Scottish Bacterial STI Reference Laboratory	All patients with gonorrhoea	All cultures,and sample of NAAT patients where culture not available	Primary care, sexual health services
Switzerland (29)	Other	Subnational – North-Eastern Switzerland and Zurich	Patients with symptomatics of gonorrhoea	NA	Not specified
**WHO South East Asia Region**					
India (30)	Other	Subnational – STD clinics in New Delhi (capital city), Pune, Mumbai, Hyderabad and Secuderabad	Patients with symptomatics of gonorrhoea	NA	STD clinics
Nepal (31)	Other	National – 13 laboratories in hills and at ground level, none in mountainous areas	NA	Consecutive	Not specified
**WHO Western Pacific Region**					
Australia (32)	Comprehensive	National	All patients with gonorrhoea	All cultures and sample of NAAT+ patients where culture not available	Primary care, sexual health services, hospitals, private sites
China (33)	Sentinel	Subnational - Clinics in 9 provinces (Guangdong, Guangxi, Hainan, Zhejiang, Sichuan, Tianjin, Xinjiang, Shanghai, Chongquing)	All patients with gonorrhoea	Culture following positive risk assessment	Local dermatology and/or STD clinics
Hong Kong Special Administrative Region (34)	Other	National	All patients	NA	STD clinics
Japan (35)	Other	National - cooperating medical institutions in the regions of Hokkaido, Miyagi, Tokyo, Saitama, Shizuoka, Aichi, Gifu, Ishikawa, and Shiga	All patients with gonorrhoea	NA	Medical institutions
Korea (36)	Other	National – 35 urological clinics and other sites	Men or women with urethritis (male or female) and female sex workers	NA	Urological clinics, public health centres, secondary care hospitals and tertiary care hospitals
New Zealand (37, 38)	Comprehensive	National – data from laboratories in 10 out 17 district health boards	NA	NA<br>Enhanced culturing for 2014-15 survey	Not specified
Thailand (39)	Sentinel	Subnational -2 sentinel sites and 2 reference laboratories in Bangkok, Thailand	Patients with symptomatics of gonorrhoea	Continually enrols persons with confirmed gonorrhoea e.g. in Thailand all symptomatic male patients with urethritis	1 hospital, 1 community clinic

Supplementary Table S2b System type, coverage, target population, sampling frame and collection sites for countries included in multi-country World Health Organisation Multi-country Gonococcal Antimicrobial Surveillance Programs (GASP)

Country/Region	System Type	Coverage	Population	Sampling[2]	Where isolates are collected
**Europe (40-43)**	Sentinel	27/30 countries[1]	NA		
Austria	Other	National		Consecutive	GPs, gynaecological clinics, urologists and sex worker monitoring
Belgium	Comprehensive	National		Consecutive	GPs, hospitals, STI clinics, gynaecological clinics
Croatia	Sentinel	National		Consecutive	STI clinics, DV clinics, GPs, hospitals
Cyprus	Unknown	National		Unknown	DV and urology clinics
Czech Republic	Sentinel	Not available		Consecutive	DV clinic
Denmark	Comprehensive	National		Consecutive	STI clinics, DV clinics, GPs, hospitals
Estonia	Other	National		Consecutive	All
Finland		Not available		Consecutive	Not available
France	Sentinel	National		Consecutive	GPs, STI clinics and hospitals
Germany	Other	Not available		Consecutive	Medical practices, outpatients, hospital laboratories, public health departments and STI ambulances
Greece	Other	National		Consecutive	STI clinics and general hospitals
Hungary	Sentinel	Not available		Selectively	STI clinics
Iceland	Comprehensive	National		Consecutive	STI clinics, DV clinics, GPs, hospitals, private practitioners
Ireland	Other	National		Consecutive	STI clinic and GPs
Italy	Comprehensive	Not available		Consecutive	STI clinics, hospital, university/hospital microbiology units, DV clinics
Latvia	Other	National		Consecutive	STI clinics
Luxembourg	Other	National		Consecutive	GPs, hospitals, urology and family planning clinics
Malta	Comprehensive	National		Selectively	STI clinic, GPs and hospitals
Netherlands	Sentinel	Subnational		Consecutive	STI clinic
Norway	Unknown	National		Consecutive	STI clinics, GPs
Poland	Other	Not available		Consecutive	STI clinic
Portugal	Other	National		Consecutive	STI clinics, DV clinics, GPs, hospitals, urology and gynaecology clinics
Slovakia	Comprehensive	Not available		Consecutive	DV, urology and gynaecology clinics
Slovenia	Other	Not available		Consecutive	DV and STI clinics
Spain	Sentinel	National		Consecutive	STI clinics and hospitals
Sweden	Comprehensive	National		Consecutive	STI clinics
United Kingdom	Sentinel	National		Consecutive	GUM/STI clinics, GPs and outpatients
**Latin America and the Caribbean (44)**	Other	Various - 23 out 41 countries reported data at some level (national, local, ongoing periodic) between 1990 and 2011. Some countries have established national GASP networks (e.g. Argentina, Chile) while others reported from single major urban areas (Columbia, Venezuela).	NA	NA	Not specified
**South-East Asia (45)**	Other	6 countries out of 11 SEAR countries, 18 focal laboratories (4 Bhutan, 1 Indonesia, 10 India, 1 Myanmar, 1 Sri Lanka, 1 Thailand)	Various	NA	Mixed
Bhutan		Subnational - Thimpu, Gelephu, Mongar, Phuentsholing	STD clinic attendees	As above	STI clinics
India		Subnational - Delhi (4), Pune, Chennai, Hyderabad, Kolkata, Nagpur, Vadodra	STD clinic attendees, MSM, FSW	As above	STI clinics, other

Indonesia		Not available	Referred by private clinics (community-based)	As above	Private clinics
Myanmar		Not available	FSW	As above	As above
Sri Lanka		Not available	STD clinic attendees	As above	STI clinics
Thailand		Not available	Patients with STDs, FSW, MSM	As above	As above
Western Pacific (46)	Other	12 out of 27 WPRO countries	Primarily patients attending STI clinics	Primarily unselected	Not specified
Global (47)	Other	52 out of 193 WHO member states in 2014	Various	Various	Not specified

Note: 1. Laboratories chosen by member states based on local epidemiology and convenience (e.g. laboratory with links to large STI clinic).

2. Collection September to November 2018, except for the United Kingdom (UK), which collected isolates between July and September 2018 to coincide with GRASP.

Supplementary Table S3: Anatomical site, reported cases and data collection variables in included surveillance systems

Country/Region	Anatomical sites				Isolates (n)	Timeframe	% NG cases	Patient characteristics	Linked
	Male		Female						
	Anogenital	Non-anogenital	Anogenital	Non-Anogenital					
Ghana (2)	NA	NA	NA	NA	13	2012-2013	NA	Age and sex	No
Kenya (3)	No	No	Yes	NA	238	2012-2015	NA	NA	NA
South Africa (4, 5)	Yes	No	Yes	No	4224[1]	2008-2015	NA	NA	NA
Zimbabwe (6)	Yes	No	No	No	102	2015-2016	NA	Age	No
Argentina (8)	NA	NA	NA	NA	1987	2009-2013	NA	NA	NA
Côte d'Ivoire (7)	Yes	No	Yes	No	212	2014-2017	NA	age, gender, sexual orientation, symptoms	
Brazil (9)	Yes	No	No	No	550	2015-2016	NA	No complete or reliable clinical / epidemiological data available	No
Canada (10, 11, 48)	Yes	Yes	Yes	Yes	4538	2016	19.1%	Age and sex	No
USA (12-14) (GISP)	Yes	No	No	No	5160	2018	1.5	Age, race/ethnicity, sex, partner sex, previous diagnoses of gonorrhoea, HIV status, recent travel, and antimicrobial gonorrhoea treatment.	Yes – abstracted from medical records by clinic staff and submitted each month using
USA (eGISP)(15, 16)	Yes	Yes	Yes	Yes	280	2017-2019	NA	In addition to above: Anatomic site of isolate collection, meningococcal vaccination history, and possible Neisseria meningitidis isolate	Yes- eGISP patient ID link laboratory results and epidemiologic data
USA (SURRG)(15)	Yes	Yes	Yes	Yes	1664 (in Indiana)	2017-2019	NA	In addition to GISP, Sexual history	Yes
Morocco (17)	Yes	No	No	No	72	2009	NA	Age	No
Austria (18)	Yes	Yes	Yes	Yes	3584	2010-2014	NA	Sample collection site (genital, rectal, pharyngeal), gender, age, sexual orientation, known gonococcal infections in the past, and possible coinfections with other STIs.	No - questionnaire for anonymized data given to sites
Belarus (19)	Yes	No	Yes	No	193	2010-2013	NA	Age and sex	NA
England and Wales (GRASP) (20, 21)	Yes	Yes	Yes	Yes	1284	2016	3.5	Age, sex, patient residence, ethnicity, symptoms, previously diagnosed with NG, concurrent STI, site of infection, STI status, total partners in last 3 months, sex abroad, sexual orientation, plus PHE reporting form: date of attendance, attendance type, gender, age, ethnicity, COB, sexual orientation, concurrent STIs. From medical	Yes - to PHE reporting form

Country/Region	Anatomical sites				Isolates (n)	Timeframe	% NG cases	Patient characteristics	Linked
	Male		Female						
	Anogenital	Non-anogenital	Anogenital	Non-Anogenital					
								records - DOB, previous NG diagnosis, diagnostic methods used, sites of infection and symptom presence, antibiotic treatment for NG/CT, TOC, sexual contacts in UK or abroad	
England and Wales (SGSS) (49, 50)	NA	NA	NA	NA	17099	2016	47.2	Age and sex	No
France (Rénago only) (24)	Yes	Yes	Yes	Yes	8649	2001-12	NA	sex, age, date of sample, anatomical site of sample, presence of symptoms, co-infection with another STI, probable country of infection, partner diagnosis, clinical site, specialisation of treating physician, type of laboratory and geographical area to which it belongs. The sexual orientation of the patient is not reported.[4]	No
Germany (25)	Yes	Yes	Yes	Yes	1654	2014-2015	NA	Site, district code, gender, year of birth is transmitted electronically with information on test results etc to RKI	No
Italy (26)	Yes	Yes	Yes	Yes	1688	2009-2016	NA	unlinked anonymous demographic, clinical and laboratory data received - include age, gender, sexual orientation, citizenship, site of infection, HIV status, previous diagnosis	No
Netherlands (27)	Yes	Yes	Yes	Yes	11678	2007-15	41.8	Age, sex, sexual orientation, sexual behaviours, previous STI infection and co-infection.	No – obtained from patient file
Russia (28)	Yes	No	Yes	No	5038	2005-2016	NA	NA	NA
Scotland (22, 23)	NA	NA	NA	NA	3168	2018	46.9	Sex	NA
Switzerland (29)	Yes	Yes	Yes	Yes	318	1990, 2000-2012	NA	Specimen source, date specimen obtained, sex, age, and specimen site (2005 onwards)	NA
India (30)	Yes	No	Yes	No	124	2013-2016	NA	Age, sex, marital status, education, employment, sexual history (including FSW or MSM), past STIs, past treatment	NA
Nepal (31)	NA	NA	NA	NA	181	1999-2012	NA	NA	NA
Australia (32)	Yes	Yes	Yes	Yes	9668	2019	28	Sex, country of acquisition, site of isolation, state of residence	No
China (33)	Yes	No	Yes	No	3849[2]	2013-2016	0.8	Age, sex, sexual orientation, site, ethnicity, previous gonococcal infection	Yes – extracted from outpatient records
Hong Kong Special Administrative Region (34)	Yes	No	Yes	No	947	2010	NA	Clinical and epidemiological information including sexual exposure history	No – collected from patients attending STD clinics

Country/Region	Anatomical sites				Isolates (n)	Timeframe	% NG cases	Patient characteristics	Linked
	Male		Female						
	Anogenital	Non-anogenital	Anogenital	Non-Anogenital					
Japan (35)	Yes	Yes	Yes	Yes	2471	2000-2015	NA	NA	NA
Korea (36)	NA	NA	NA	NA	210	2011-2013	NA	Prescription data from Korean Health Insurance and Assessment Service, not linked to patient	No
New Zealand (37, 38)	Yes	Yes	Yes	Yes	667	2014	22.0	Date of birth, sex, specimen site	No
Thailand (39)	Yes	No	No	No	590	NA	NA	Behavioural and clinical data e.g. demographics, prior antibiotic use, sexual behaviour history and treatment are collected on a case abstraction form	No
Europe (40-43, 51, 52)	Yes	Yes	Yes	Yes	3299	2018		Date specimen obtained, specimen site, age, gender, mode of transmission, sexual orientation, previously diagnosed with NG and/or concurrent STI during current episode, place of residence, clinical service type, HIV status, probable country of infection, diagnostic test and treatment used. [5]	Yes
Austria					267	2018	NA		
Belgium					180	2018	5		
Croatia					10	2018	75		
Czech Republic					95	2018	6		
Cyprus					5	2018	NA		
Denmark					114	2018	6		
Estonia					7	2018	2		
Finland					168	2018	NA		
France					109[3]	2018	1		
Germany					201	2018	NA		
Greece					83	2018	NA		
Hungary					89[3]	2018	8		
Iceland					45	2018	37		
Ireland					200	2018	5		
Italy					100	2018	13		
Latvia					5	2018	5		
Luxembourg					1	2018	222		
Malta					25	2018	34		
Netherlands					402[3]	2018	4		
Norway					126	2018	10		
Poland					73	2018	18		
Portugal					122	2018	40		

Country/Region	Anatomical sites				Isolates (n)	Timeframe	% NG cases	Patient characteristics	Linked
	**Male**		**Female**						
	**Anogenital**	**Non-anogenital**	**Anogenital**	**Non-Anogenital**					
Slovakia					77	2018	40		
Slovenia					155	2018	131		
Spain					189	2018	5		
Sweden					200	2018	6		
United Kingdom					251	2018	1		
Latin America and the Caribbean (44)	NA	NA	NA	NA	21592	1990-2011	NA	NA	NA
South-East Asia (45)	NA	NA	NA	NA	4675	2009-2012	NA	Sex	NA
Bhutan					991	2009-2012	NA		
India					424	2009-2012	NA		
Indonesia					24	2010-2012	NA		
Myanmar					40	2011	NA		
Sri Lanka					233	2009-2012	NA		
Thailand					2949	2009-2012	NA		
Western Pacific(46)	NA	NA	NA	NA	8484	2011	NA	NA	NA
Australia					4106	2011	NA		
Brunei Darussalem					295	2011	NA		
China					1349	2011	NA		
Fiji					252	2011	NA		
Hong Kong SAR					1225	2011	NA		
Japan					441	2011	NA		
Republic of Korea					64	2011	NA		
New Caledonia					166	2011	NA		
New Zealand					317	2011	NA		
The Philippines					34	2011	NA		
Singapore					160	2011	NA		
Vietnam					75	2011	NA		
Global (47)	NA	NA	NA	NA	NA	NA	NA	NA	NA
WHO Antimicrobial Resistance Global Surveillance Report (53)	NA	NA	NA	NA	21330	Various	NA	NA	NA

1.2 geographic areas only

2.7 out of 9 provinces only

3. Sentinel epidemiological data

4.Because of the large proportion of missing data for four variables (presence of symptoms, co-infection with another STI, probable country of infection, and information on patient's partner infection status), the latter were used only for univariate analysis for descriptive purposes.

5.Note in 2015, completeness high for gender, age and site of infection, improving for mode of transmission previous NG, clinical service type, country of birth and probably country of infection, low e.g. for HIV status

#The data of % NG cases from Europe region were reported in the Euro-GASP report 2016, no data were reported for 2018; percentages above 100% suggest under-reporting of cases in epidemiological surveillance.

Supplementary Table S4: Laboratory procedures used in included surveillance systems

Country	Centralised	Agar Dilution	E-test	Beta-lactamase	NG-MAST	Disk diffusion	NAAT	Criteria	CX[1]	CIP[1]	TET[1]	SPE[1]	PEN[1]	AZ[1]	CE[1]	OTH[1]
Ghana (2)	Decentralised							NA								
Kenya (3)	NA							WHO								
South Africa (4, 5)	NA							CLSI, EUCAST for azithromycin								
Côte d'Ivoire (7)	Centralised							CISI, WHO								
Zimbabwe (6)	Centralised							CLSI, WHO								
Argentina (8)	Centralised							CLSI, EUCAST for azithromycin								
Brazil (9)	Centralised							CLSI, EUCAST for azithromycin								
Canada (10, 11, 48)	Decentralised							CLSI								
USA (GISP) (12-14)	Decentralised							CLSI								
USA (eGISP)(15, 16)	Decentralised							CLSI								
USA (SURRG)(15)	Decentralised							CLSI								
Morocco (17)	Decentralised							CLSI								
Austria (18)	Centralised							CLSI, EUCAST								
Belarus (19)	Centralised							EUCAST								

Country	Centralised	Agar Dilution	E-test	Beta-lactamase	NG-MAST	Disk diffusion	NAAT	Criteria	CX[1]	CIP[1]	TET[1]	SPE[1]	PEN[1]	AZ[1]	CE[1]	OTH[1]
England and Wales (GRASP) (20, 21)	Centralised							EUCAST								
England and Wales (SGSS) (49, 50)	Decentralised							EUCAST								
Scotland (22, 23)	Centralised							EUCAST								
France (Rénago only) (24)	Centralised							EUCAST								
Germany (25)	Decentralised							EUCAST								
Italy (26)	Centralised							EUCAST								
Netherlands (27)	Centralised							EUCAST								
Russia (28)	Centralised							CLSI, EUCAST for azithromycin								
Switzerland (29)	Centralised							EUCAST								
India (30)	Decentralised							CLSI								
Nepal (31)	Decentralised							NA								
Thailand (39)	Decentralised							NA								
Australia (32)	Decentralised							CDS								
China (33)	Decentralised							CLSI								
Hong Kong SAR (34)	Centralised							CLSI, EUCAST for azithromycin								
Japan (35)	Centralised							CLSI, EUCAST for azithromycin								
Korea (36)	Centralised							EUCAST, CLSI for cefpodoxime								
New Zealand (37, 38)	Decentralised							CLSI, EUCAST for azithromycin								
Europe (40-43, 52)	Decentralised/centralised							EUCAST								
Austria	Decentralised							EUCAST								

Country	Centralised	Agar Dilution	E-test	Beta-lactamase	NG-MAST	Disk diffusion	NAAT	Criteria	CX[1]	CIP[1]	TET[1]	SPE[1]	PEN[1]	AZ[1]	CE[1]	OTH[1]
Belgium	Decentralised							EUCAST								
Croatia	Centralised							EUCAST								
Cyprus	Decentralised							EUCAST								
Czech Republic	Centralised							EUCAST								
Denmark	Decentralised							EUCAST								
Estonia	Centralised							EUCAST								
Finland	Decentralised							EUCAST								
France	Decentralised							EUCAST								
Germany	Centralised							EUCAST								
Greece	Decentralised							EUCAST								
Hungary	Centralised							EUCAST								
Iceland	Decentralised							EUCAST								
Ireland	Decentralised							EUCAST								
Italy	Decentralised							EUCAST								
Latvia	Centralised							EUCAST								
Malta	Decentralised							EUCAST								
Netherlands	Decentralised							EUCAST								
Norway	Decentralised							EUCAST								
Poland	Centralised							EUCAST								
Portugal	Decentralised							EUCAST								
Slovakia	Centralised							EUCAST								
Slovenia	Decentralised							EUCAST								
Spain	Decentralised							EUCAST								
Sweden	Decentralised							EUCAST								
United Kingdom	Decentralised							EUCAST								
Luxembourg	Centralised							EUCAST								
Latin America and the Caribbean (44)	Decentralised							CLSI except azithromycin breakpoint based on "established criteria"								

Country	Centralised	Agar Dilution	E-test	Beta-lactamase	NG-MAST	Disk diffusion	NAAT	Criteria	CX[1]	CIP[1]	TET[1]	SPE[1]	PEN[1]	AZ[1]	CE[1]	OTH[1]
South-East Asia (45)	Decentralised							CLSI, CDS								
Bhutan	Decentralised							CLSI, CDS								
India	Decentralised							CLSI, CDS								
Indonesia	Centralised							CLSI								
Myanmar	Centralised							CDS								
Sri Lanka	Centralised							CDS								
Thailand	Centralised							CLSI, CDS								
Western Pacific (46)	Decentralised	NA	NA	NA	NA	NA		NA								
Australia	Decentralised															
Brunei Darussalem	NA															
China	NA															
Fiji	NA															
Hong Kong SAR	NA															
Japan	NA															
Republic of Korea	NA															
New Caledonia	NA															
New Zealand	NA															
The Philippines	NA															
Singapore	NA															
Vietnam	NA															
Global (47)	Decentralised	NA	NA	NA	NA	NA		NA								

*CX – ceftriaxone, CIP – ciprofloxacin, TET- tetracycline, SPE-spectinomycin, PEN-penicillin, AZ – azithromycin, CE – Cefixime, OTH – Other

Cip/Pen/Cro/AZI are the GASP ABS – so all GASP labs generally test and report to the WHO

1. World Health Organization. National antimicrobial resistance surveillance systems and participation in the Global Antimicrobial Resistance Surveillance System (GLASS): Core componets checklist and questionnaire. Geneva; WHO; 2016. Contract No.: WHO/DGO/AMR/2016.5.
2. Duplessis C, Puplampu N, Nyarko E, Carroll J, Dela H, Mensah A, et al. Gonorrhea surveillance in Ghana, Africa. Military medicine. 2015;180(1):17-22.
3. Omolo MJ, Pole L, Mwangi I, Kimani J, Anzala O, Oloo J, et al. P2.30 Survey of antimicrobial resistance in clinical neisseria gonorrhoeae isolated over a period of four years in nairobi - kenya. Sex Transm Infect. 2017;93.
4. Kularatne R, Maseko V, Gumede L, Radebe F, Kufa-Chakezha T. P3.186 Neisseria gonorrhoeae antimicrobial resistance surveillance in johannesburg, south africa. Sexually Transmitted Infections. 2017;93(Suppl 2):A162-A.
5. Kularatne R MV, Gumede L, Radebe F, Kufa-Chakezha T,. Neisseria Gonorrheae anitmicrobial resistance surveillance in Gauteng Province, South Africa. Communicable Diseases Surveillance Bulletin. 2016;14 (3): 56-64.
6. Latif AS, Gwanzura L, Machiha A, Ndowa F, Tarupiwa A, Gudza-Mugabe M, et al. Antimicrobial susceptibility in Neisseria gonorrhoeae isolates from five sentinel surveillance sites in Zimbabwe, 2015–2016. Sexually Transmitted Infections. 2017.
7. Yeo A, Kouame-Blavo B, Kouame CE, Ouattara A, Yao AC, Gbede BD, et al. Establishment of a Gonococcal Antimicrobial Surveillance Programme, in Accordance With World Health Organization Standards, in Cote d'Ivoire, Western Africa, 2014-2017. Sex Transm Dis. 2019;46(3):179-84.
8. Gianecini R, de las Mercedes Romero M, Oviedo C, Vacchino M, Galarza P, Romero MdLM. Emergence and Spread of Neisseria gonorrhoeae Isolates With Decreased Susceptibility to Extended-Spectrum Cephalosporins in Argentina, 2009 to 2013. Sex Transm Dis. 2017;44(6):351-5.
9. Bazzo ML, Golfetto L, Gaspar PC, Pires AF, Ramos MC, Franchini M, et al. First nationwide antimicrobial susceptibility surveillance for Neisseria gonorrhoeae in Brazil, 2015-16. The Journal of antimicrobial chemotherapy. 2018.
10. Martin I, Sawatzky P, Allen V, Lefebvre B, Hoang L, Naidu P, et al. Multidrug-resistant and extensively drug-resistant Neisseria gonorrhoeae in Canada, 2012-2016. Can Commun Dis Rep. 2019;45(2-3):45-53.
11. Gratrix J, Kamruzzaman A, Martin I, Smyczek P, Read R, Bertholet L, et al. Surveillance for Antimicrobial Resistance in Gonorrhea: The Alberta Model, 2012(-)2016. Antibiotics (Basel). 2018;7(3).
12. Kirkcaldy RD, Harvey A, Papp JR, del Rio C, Soge OO, Holmes KK, et al. Neisseria gonorrhoeae antimicrobial susceptibility surveillance - The Gonococcal Isolate Surveillance Project, 27 sites, United States, 2014. MMWR Surveillance Summaries. 2016;65(7):1-24.
13. Mann LMMPH, Kirkcaldy RDMD, Papp JRP, Torrone EAPM. Susceptibility of Neisseria gonorrhoeae to Gentamicin-Gonococcal Isolate Surveillance Project, 2015-2016. Sex Transm Dis. 2018;45(2):96.
14. US Centers for Disease Control and Prevention. Gonococcal Isolate Surveilance Program Protocol May 2016. 2016.
15. Kersh EN, Pham CD, Papp JR, Myers R, Steece R, Kubin G, et al. Expanding U.S. Laboratory Capacity for Neisseria gonorrhoeae Antimicrobial Susceptibility Testing and Whole-Genome Sequencing through the CDC's Antibiotic Resistance Laboratory Network. J Clin Microbiol. 2020;58(4).
16. Prevention CfDCa. Gonococcal Isolate Surveillance Project (GISP) and Enhanced GISP (eGISP). Atlanta, Georgia Centers for Disease Control and Prevention 2020.
17. Hancali A, Ndowa F, Bellaji B, Bennani A, Kettani A, Charof R, et al. Antimicrobial resistance monitoring in Neisseria gonorrhoeae and strategic use of funds from the Global Fund to set up a systematic Moroccan gonococcal antimicrobial surveillance programme. Sex Transm Infect. 2013;89(Suppl 4):i24-i7.
18. Stary A, Heller-Vitouch C, Binder M, Geusau A, Stary G, Rappersberger K, et al. Gonococcal infections in Austria: a long-term observation of prevalence and resistance profiles from 1999 to 2014. J. 2015;13(11):1136-45.
19. Lebedzeu F, Golparian D, Titov L, Pankratava N, Glazkova S, Shimanskaya I, et al. Antimicrobial susceptibility/resistance and NG-MAST characterisation of Neisseria gonorrhoeae in Belarus, Eastern Europe, 2010-2013. BMC Infectious Diseases. 2015;15:29.

20. Clifton S, Bolt H, Mohammed H, Town K, Furegato M, Cole M, et al. Prevalence of and factors associated with MDR Neisseria gonorrhoeae in England and Wales between 2004 and 2015: analysis of annual cross-sectional surveillance surveys. Journal of Deaf Studies & Deaf Education. 2018;23(2):923-32.
21. Health Protection Agency. Gonococcal Resistance to Antimicrobials Surveillance Programme (GRASP) Action Plan for England and Wales: Informing the Public Health Response. 2013.
22. Health Protection Scotland and NHS Lothian. Scottish Bacterial Sexually Transmitted Infections Reference Laboratory User Manual 2015. 2015.
23. Jill Shepherd, Lesley Wallace, Martin McHugh, Beth Cullen, Cameron R, Goldberg D. Gonococcal antibiotic surveillance in Scotland (GASS): prevalence, patterns and trends in 2018. National Services Scotland; 2019.
24. La Ruche G, Goubard A, Bercot B, Cambau E, Semaille C, Sednaoui P. Gonococcal infections and emergence of gonococcal decreased susceptibility to cephalosporins in France, 2001 to 2012. Euro Surveillance: Bulletin Europeen sur les Maladies Transmissibles = European Communicable Disease Bulletin. 2014;19(34):28.
25. Buder S, Dudareva S, Jansen K, Loenenbach A, Nikisins S, Sailer A, et al. Antimicrobial resistance of Neisseria gonorrhoeae in Germany: low levels of cephalosporin resistance, but high azithromycin resistance. BMC Infect Dis. 2018;18(1):44.
26. Stefanelli P, Vescio MF, Landini MP, Dal Conte I, Matteelli A, Cristaudo A, et al. Time trend analysis (2009-2016) of antimicrobial susceptibility in Neisseria gonorrhoeae isolated in Italy following the introduction of the combined antimicrobial therapy. PLoS ONE. 2017;12(12):e0189484.
27. Hofstraat SH, Gotz HM, van Dam AP, van der Sande MA, van Benthem BH. Trends and determinants of antimicrobial susceptibility of Neisseria gonorrhoeae in the Netherlands, 2007 to 2015. Euro Surveill. 2018;23(36).
28. Kubanov A, Solomka V, Plakhova X, Chestkov A, Petrova N, Shaskolskiy B, et al. Summary and Trends of the Russian Gonococcal Antimicrobial Surveillance Programme, 2005 to 2016. J Clin Microbiol. 2019;57(6).
29. Kovari H, de Melo Oliveira MD, Hauser P, Lauchli S, Meyer J, Weber R, et al. Decreased susceptibility of Neisseria gonorrhoeae isolates from Switzerland to Cefixime and Ceftriaxone: antimicrobial susceptibility data from 1990 and 2000 to 2012. BMC Infectious Diseases. 2013;13:603.
30. Kulkarni SV, Bala M, Muqeeth SA, Sasikala G, Nirmalkar AP, Thorat R, et al. Antibiotic susceptibility pattern of Neisseria gonorrhoeae strains isolated from five cities in India during 2013-2016. Journal of medical microbiology. 2018;67(1):22-8.
31. Malla S, Dumre SP, Geeta S, Palpasa K, Bhupraj R, Anowar H, et al. The challenges and successes of implementing a sustainable antimicrobial resistance surveillance programme in Nepal. BMC Public Health. 2014;14(269).
32. Lahra MM, Enriquez RP, George CRR. Australian Gonococcal Surveillance Programme Annual Report, 2019. Communicable Diseases Intelligence 2020;44.
33. Yin YP, Han Y, Dai XQ, Zheng HP, Chen SC, Zhu BY, et al. Susceptibility of Neisseria gonorrhoeae to azithromycin and ceftriaxone in China: A retrospective study of national surveillance data from 2013 to 2016. Plos Medicine. 2018;15(2).
34. Lo JY, Ho KM, Lo AC. Surveillance of gonococcal antimicrobial susceptibility resulting in early detection of emerging resistance. Journal of Antimicrobial Chemotherapy. 2012;67(6):1422-6.
35. Yasuda M, Hatazaki K, Ito S, Kitanohara M, Yoh M, Kojima M, et al. Antimicrobial Susceptibility of Neisseria gonorrhoeae in Japan from 2000 to 2015. Sex Transm Dis. 2017;44(3):149-53.
36. Lee H, Unemo M, Kim H, Seo Y, Lee K, Chong Y. Emergence of decreased susceptibility and resistance to extended-spectrum cephalosporins in Neisseria gonorrhoeae in Korea. Journal of Antimicrobial Chemotherapy. 2015;70(9):2536-42.
37. Institute for Environmental Science and Research Limited. Antimicrobial resistance and molecular epidemiology of Neisseria gonorrhoeae in New Zealand, 2014-15. 2015.

38. Institute for Environmental Science and Research Limited. Sexually Transmitted Infections in New Zealand: Annual Surveillance Report 2014. Porirua, New Zealand; 2015

39. Sirivongrangson P, Girdthep N, Sukwicha W, Buasakul P, Tongtoyai J, Weston E, et al. The first year of the global Enhanced Gonococcal Antimicrobial Surveillance Programme (EGASP) in Bangkok, Thailand, 2015-2016. PloS one. 2018;13(11):e0206419.

40. European Centre for Disease Control and Prevention. Response plan to control and manage the threat of multi-drug resistant gonorrhoea in Europe. . 2012.

41. European Centre for Disease Control and Prevention. Gonococcal antimicrobial susceptibility surveillance in Europe, 2015. 2017.

42. Cole MJ, Spiteri G, Jacobsson S, Woodford N, Tripodo F, Amato-Gauci AJ, et al. Overall Low Extended-Spectrum Cephalosporin Resistance but high Azithromycin Resistance in Neisseria gonorrhoeae in 24 European Countries, 2015. BMC Infectious Diseases. 2017;17(1):617.

43. Spiteri G, Cole M, Unemo M, Hoffmann S, Ison C, Laar Mvd. The European Gonococcal Antimicrobial Surveillance Programme (Euro-GASP) - a sentinel approach in the European Union (EU)/European Economic Area (EEA). (Special Issue: Gonococcal antimicrobial resistance.). Sex Transm Infect. 2013;89(Suppl. 4).

44. Dillon J-AR, Trecker MA, Thakur SD, Fiorito S, Galarza P, Trigoso Carvallo ME, et al. Two decades of the gonococcal antimicrobial surveillance program in South America and the Caribbean: challenges and opportunities. Sex Transm Infect. 2013;89(Suppl 4):iv36-iv41.

45. Bala M, Kakran M, Singh V, Sood S, Ramesh V, Members of WHOGSN. Monitoring antimicrobial resistance in Neisseria gonorrhoeae in selected countries of the WHO South-East Asia Region between 2009 and 2012: a retrospective analysis. Sex Transm Infect. 2013;89 Suppl 4:iv28-iv35.

46. Lahra MM, Lo Y, Whiley DM. Gonococcal antimicrobial resistance in the Western Pacific Region. (Special Issue: Gonococcal antimicrobial resistance.). Sex Transm Infect. 2013;89(Suppl. 4).

47. Wi T, Lahra MM, Ndowa F, Bala M, Dillon JR, Ramon-Pardo P, et al. Antimicrobial resistance in Neisseria gonorrhoeae: Global surveillance and a call for international collaborative action. PLoS medicine. 2017;14(7):e1002344.

48. Martin I, Sawatzky P, Liu G, Allen V, Lefebvre B, Hoang L, et al. Decline in Decreased Cephalosporin Susceptibility and Increase in Azithromycin Resistance in Neisseria gonorrhoeae, Canada. Emerging infectious diseases. 2016;22(1):65-7.

49. Public Health England. Antimicrobial resistance in Neisseria gonorrhoeae in England and Wales: Key findings from the Gonococcal Resistance to Antimicrobials Surveillance Programme (GRASP 2018). London; 2019.

50. Public Health England. Surveillance of antimicrobial resistance in Neisseria gonorrhoeae

Key findings from the Gonococcal Resistance to Antimicrobials Surveillance Programme (GRASP). 2016.

51. Day MJ, Spiteri G, Jacobsson S, Woodford N, Amato-Gauci AJ, Cole MJ, et al. Stably high azithromycin resistance and decreasing ceftriaxone susceptibility in Neisseria gonorrhoeae in 25 European countries, 2016. BMC Infect Dis. 2018;18(1):609.

52. European Centre for Disease Prevention and Control. Gonococcal antimicriobial susceptibility surveillance in Europe-Results summary 2018. Stockholm ECDC; 2020.

53. World Health Organisation. Antimicrobial Resistance Global Surveillance Report. Geneva; 2014.
